# Efficient Removal of Platelets from Peripheral Blood Progenitor Cell Products Using a Novel Micro-Chip Based Acoustophoretic Platform

**DOI:** 10.1371/journal.pone.0023074

**Published:** 2011-08-09

**Authors:** Josefina Dykes, Andreas Lenshof, Ing-Britt Åstrand-Grundström, Thomas Laurell, Stefan Scheding

**Affiliations:** 1 Clinical Immunology and Transfusion Medicine, Regional and University Laboratories, Lund, Sweden; 2 Stem Cell Center, Institution of Laboratory Medicine, Lund University, Lund, Sweden; 3 Measurement Technology and Industrial Electrical Engineering, Lund University, Lund, Sweden; 4 Department of Hematology, University Hospital Skane, Lund, Sweden; 5 Biomedical Engineering, Dongguk University, Seoul, Korea; University of Pittsburgh, United States of America

## Abstract

**Background:**

Excessive collection of platelets is an unwanted side effect in current centrifugation-based peripheral blood progenitor cell (PBPC) apheresis. We investigated a novel microchip-based acoustophoresis technique, utilizing ultrasonic standing wave forces for the removal of platelets from PBPC products. By applying an acoustic standing wave field onto a continuously flowing cell suspension in a micro channel, cells can be separated from the surrounding media depending on their physical properties.

**Study Design and Methods:**

PBPC samples were obtained from patients (n = 15) and healthy donors (n = 6) and sorted on an acoustophoresis-chip. The acoustic force was set to separate leukocytes from platelets into a target fraction and a waste fraction, respectively. The PBPC samples, the target and the waste fractions were analysed for cell recovery, purity and functionality.

**Results:**

The median separation efficiency of leukocytes to the target fraction was 98% whereas platelets were effectively depleted by 89%. PBPC samples and corresponding target fractions were similar in the percentage of CD34+ hematopoetic progenitor/stem cells as well as leukocyte/lymphocyte subset distributions. Median viability was 98%, 98% and 97% in the PBPC samples, the target and the waste fractions, respectively. Results from hematopoietic progenitor cell assays indicated a preserved colony-forming ability post-sorting. Evaluation of platelet activation by P-selectin (CD62P) expression revealed a significant increase of CD62P+ platelets in the target (19%) and waste fractions (20%), respectively, compared to the PBPC input samples (9%). However, activation was lower when compared to stored blood bank platelet concentrates (48%).

**Conclusion:**

Acoustophoresis can be utilized to efficiently deplete PBPC samples of platelets, whilst preserving the target stem/progenitor cell and leukocyte cell populations, cell viability and progenitor cell colony-forming ability. Acoustophoresis is, thus, an interesting technology to improve current cell processing methods.

## Introduction

Hematopoietic stem cell transplantation is a well-established therapy for haematological malignancies and other diseases [1]–[3]. Currently, peripheral blood progenitor cells (PBPC), which can be collected from the blood after a mobilization treatment, are used for the majority of transplantations [4]–[6]. Standard apheresis technology utilizes size and density centrifugation in a continuous-flow procedure to separate the leukocyte population from whole blood while the remaining blood components are returned to the donor [7], [8].

Administration of hematopoietic growth factors, which is required for effective progenitor cell mobilization from the bone marrow into the blood, has been reported to decrease platelet counts in healthy donors before apheresis [9]. Furthermore, an unwanted and difficult to avoid side effect of current centrifugation-based PBPC apheresis is the excessive collection of platelets which leads to an additional, potentially clinically significant depletion of donor platelet levels and, consequently, an increased bleeding risk [10]–[13]. In addition, excessive platelet contamination of PBPC leads to difficulties in PBPC processing and may have a negative impact on the performance (yield and purity) of cell product manipulation, such as selection or depletion of specific cell subsets [14], [15]. Therefore, removal of platelets from PBPC for retransfusion to the donor would considerably improve both, donor safety as well as PBPC product quality.

Microfluidic devices have shown great potential in the field of complex biofluids such as blood [16]. The special conditions present in the micro domain enable multiple attractive features such as small volumes, laminar flow and fast reaction times [17]. This can be utilized in applications where cells can be manipulated in continuous flow systems [18] or retained for further operations using external forces or pure microfluidic phenomena [19].

**Figure 1:**
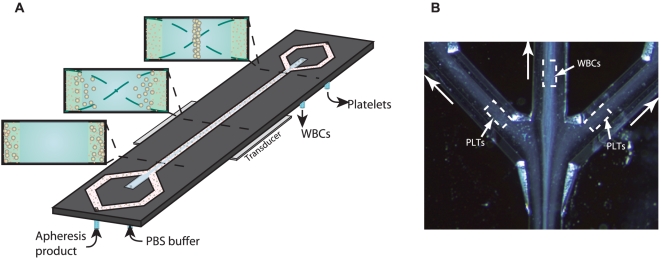
Acoustophoresis chip design. **A**) Schematic diagram of the acoustophoresis chip. Apheresis sample enters the channel from the side channels. Wash buffer enters the chip from the central inlet. The transducer generates an acoustic standing wave between the channel walls and the suspended cells are moved by the acoustic force into the pressure node located in the centre of the channel. As the acoustic force on a cell is size dependent, the larger leukocytes experience a higher radiation force than the smaller platelets. **B**) Microscope image of the separation process taken at the trifurcation. The larger leukocytes (WBC) have been centered in the channel by the ultrasonic standing wave and are collected in the central outlet, while the smaller platelets (PLT) are less acoustically affected and exit through the side branches. A movie of the platelet separation process is provided in the supplementary material.

The use of acoustic forces has recently emerged as a non contact and label free method of cell manipulation [20]. When cells or particles are subjected to an acoustic standing wave field they are exposed to radiation forces, which will induce a movement of the particle. Depending on the physical properties of the particles versus the surrounding medium, the particles are moved either to the pressure node or to the anti node of the sound field. By applying the standing wave field over a micro channel in which a suspension is flowing, the acoustic field acts as a filter which produces bands of particles located at the positions of the pressure nodes. Because of the laminar flow the particles keep their position in the band even after they have passed through the sound field, which enables separation of the particles from the surrounding medium [21]. By using a standing wave of half a wavelength, suspended particles will gather in a band located in the center of the micro channel (Figure 1A). If the separation channel ends in a trifurcation, the particles will exit through the center branch while excess fluid exits through the side branches [22].

The laminar flow also enables medium switching as the acoustic force can be used to move particles from one suspension into a medium flowing in parallel [23]. This can be used to purify samples by transferring the particles into a clean buffer from a contaminated buffer [24], [25].

The acoustic force on a particle is also size-dependent as the force is proportional to the volume. This can be utilized as a fractionation step as larger particles are transported to the pressure node faster than smaller ones. By tuning the acoustic power and the flow rate it becomes possible to sort out the larger particles [26], [27].

A combination of the medium switching and the size dependent fractionation mentioned above is used in the current study in which we investigated the performance of the acoustophoresis technique for platelet removal, aiming to efficiently deplete PBPC samples of intact platelets whilst preserving the composition and functionality of the collected target cells.

## Results

### Acoustophoretic cell separation

**Figure 2:**
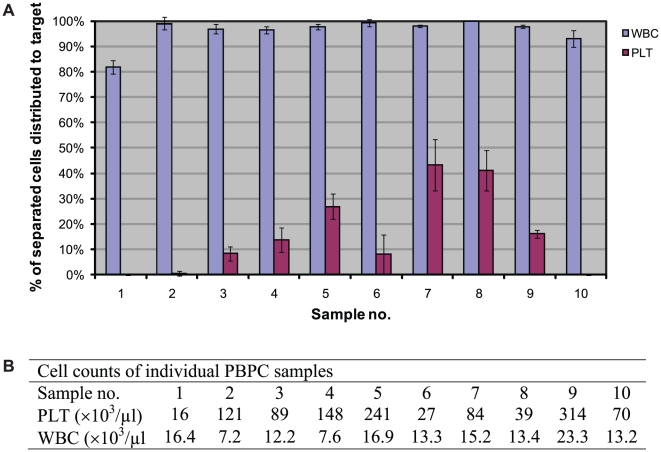
Separation efficiency of individual PBPC samples. **A**) The relative numbers (expressed as %) of separated WBC and platelets, respectively, which were distributed to the target fraction are given as the mean ± SD of 6–12 sequentially collected target portions of each PBPC sample. **B**) The WBC and PLT counts of individual PBPC samples diluted 1∶5 before separation. Samples were obtained from 2 healthy donors (sample no. 5 and 9) and 8 patients (multiple myeloma, n = 1; non Hodgkin lymphoma, n = 4; mantle cell lymphoma, n = 1; testicular cancer, n = 1; or multiple sclerosis, n = 1).

PBPC samples were obtained from 2 healthy donors and 8 patients for evaluation of acoustophoretic cell separation efficiency. For each PBPC sample, 6–12 sample portions of the separated target and waste fraction, respectively, were sequentially collected for analysis. (Table 1). The median separation efficiency of white blood cells (WBC), i.e. the median relative number of separated WBC that was collected in the target fractions, was 97.8% (range 80–100%) and the median platelet depletion was 89.0% (range 57–100%). The separation efficiency for individual PBPC samples is presented in Figure 2. The WBC separation efficiency to the target fraction was >95% in all but two samples (sample no. 1 and 10) in which however the higher loss of WBC to the waste fraction was coupled to a complete depletion (100%) of platelets from the target fraction, which was observed in only these two samples. All but three PBPC products were separated on the day of leukapheresis and platelet depletion for these products was generally higher than 90%. Overnight storage, on the other hand, resulted in considerably lower platelet depletion as low as 70% and <60% after storage at room temperature and 4°C, respectively (sample nos. 5, 7 and 8). However, insufficient platelet depletion was also observed for two of the freshly processed samples (nos. 4 and 9) that had very high platelet concentrations (≥150×10^3^/µl).

**Table 1:** Acoustophoretic Cell Separation.†

	PBPC sample	Target	Waste
*Cell counts* ‡			
WBC (×10^3^/µl)	13.4 (7.2–23.3)	6.1 (1.9–15)	0.1 (0–0.4)
PLT (×10^3^/µl)	86.5 (16–314)	8.0 (0–61)	31.0 (0–128)
RBC (×10^6^/µl)#	<0.1	<0.1	<0.1
Lymph (×10^3^/µl)	3.1 (1.9–13.7)	1.5 (0.2–9.1)	-
*Cell numbers* §			
WBC (×10^5^)	13.4 (7.2–23.3)	7.8 (2.4–18.7)	0.2 (0–0.8)
PLT (×10^5^)	86.5 (16–314)	10.2 (0–78)	62.0 (0–256)
RBC (×10^8^)	-	-	-
Lymph (×10^5^)	3.1 (1.9–13.7)	1.9 (0.3–11.6)	-
*Distribution of separated cells*			
WBC (%)		97.8 (80–100)	2.9 (0–22)
PLT (%)		12.5 (0–54.6)	89.0 (56.7–100)

Abbreviations: WBC, white blood cells; PLT, platelets; RBC, red blood cells; Lymph, lymphocytes.

† Data are presented as the median (range) of 10 samples.

‡ The PBPC samples were diluted 1∶5 before cell count.

# The RBC count was below the detection limit of 0.1 (×10^6^/µl) given in the instrument manual [44].

§ The addition of wash buffer to the acoustophoresis-chip and the difference in flow rate between the central outlet (20 µl/min) and the side oultlet (2×20 µl/min), gave a sample dilution so that the cells of 100 µl PBPC sample were distributed to 128 µl target sample (central outlet) and 200 µl waste sample (side outlet). Cell numbers were calculated accordingly.

The separation of WBC was not influenced by storage, as reflected by a WBC separation efficiency of ≥98% to the target fractions of the stored samples.

### Leukocyte population analysis

Cytospins from PBPC samples (n = 4) were prepared before and after acoustophoresis separation for evaluation of cell morphology (Table 2). The distribution of leukocyte populations in the PBPC samples and target fractions were comparable (p>0.1).

**Table 2:** Leukocyte populations.

	PBPC sample	Target	Waste
*Morphology* †			
Granulo-/monocytes (%)	70 (60–78)	68 (50–73)	12 (0–81)
Lymphocytes (%)	17 (16–28)	20 (15–39)	51 (0–91)
Promyelocytes (%)	11 (1–12)	9 (6–20)	1 (0–1)
Blast cells (%)	2 (1–2)	2 (1–9)	3 (0–8)
*Flow cytometry analysis* ‡			
CD34 (%)	2.5 (1.5–3.4)	2.3 (1.0–3.1)	1.6 (1.1–2.8)
CD3 (%)	16 (7.2–24)	17 (9.7–24)	38 (8.3–68)
CD3/4 (%)	9 (3.8–11)	10 (4.1–12)	20 (6.5–35)
CD3/8 (%)	6 (4.9–7.6)	7 (5.0–8.1)	17 (4.9–29)
CD14 (%)	43 (39–47)	45 (40–47)	23 (1.9–40)
CD56 (%)	1.9 (1.6–6)	2.3 (1.5–5.7)	1.4 (1.6–18)
CD19 (%)	2.8 (0.5–5.2)	1.3 (0.3–2.2)	1.0 (0.3–1.6)
Viability (%)#	98 (97–99)	98 (97–99)	97 (95–98)

† Data are presented as the median (range) of 4 samples (sample no. 2–4 and 7).

‡ Data are presented as the median (range) of 3 samples (sample no. 2–4).

# Viability was determined using Propidium Iodide (PI) in flow cytometry analysis.

The median relative number of granulocytes/monocytes was 70% (range 60–78%) versus 68% (50–73%) and the median relative number of lymphocytes was 17% (range 16–28%) versus 20% (range 15–39%) in the PBPC samples and target fractions, respectively. The very small total number of leukocytes which were lost in the waste fraction comprised mainly lymphocytes (median 50%, range 0–91%).

Flow cytometry analysis of PBPC samples (n = 3) before and after acoustophoretic separation revealed a similar (p>0.1) distribution of leukocyte populations in the PBSC samples and target fractions. The median relative number of CD14 + cells was 43% (range 39–47%) versus 45% (range 40–47%), the median relative number of CD3+ cells was 16% (range 7.2–24%) versus 17% (range 9.7–24%) and the median relative number of CD34+ cells was 2.5% (range 1.5–3.4%) versus 2.3% (range 1.0–3.1%), in the PBSC samples and target fractions respectively. The few leukocytes that were detected in the waste fraction were mainly lymphocytes (median 38%, range 8.3–68%). The median viability, as measured by PI-negativity, was 98% (range 97–99%) and 98% (range 97–99%) in the PBPC samples and target fractions, respectively.

### Progenitor cell colony-forming ability

**Figure 3:**
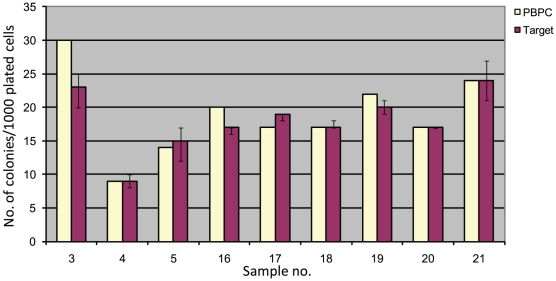
Progenitor cell colony-forming ability. The number of colonies/1,000 plated cells from 9 different PBPC samples and their corresponding target fractions are presented. The numbers of colonies are given for each single PBPC sample and as median (range) of 4–6 sequentially collected sample portions from each target fraction, respectively.

Cells from PBSC samples (n = 9) were plated in methylcellulose before and after acoustophoretic separation and evaluated for colony-forming ability (Figure 3). Results were comparable (p>0.1) in PBPC samples and target fractions, indicating a preserved clonogenic capacity post-sorting, with a median number of colonies/1,000 plated cells of 17 (range 9–30) in the PBPC samples and 17.5 (range 8–25) in the target fractions, respectively.

### Platelet activation

**Figure 4:**
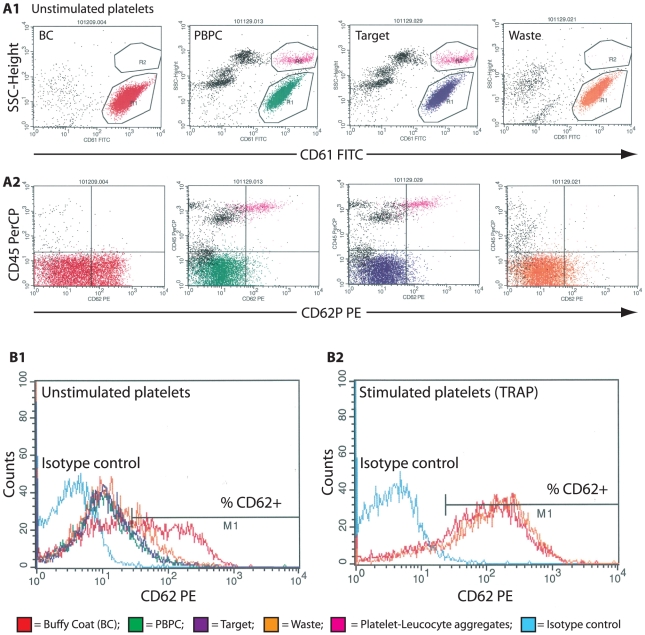
Platelet activation. Analysis of platelet activation is based on the surface expression of P-selectin (CD62P). Representative dot plots and histograms from one of five samples are shown. **A1**) The CD61+, single platelets were gated on low side scatter properties (R1). **A2**) The cells were displayed and plotted against anti-CD62P and anti-CD45 to determine the relative number of activated single platelets (CD61+/CD62P+) and to confirm the exclusion of platelet-leukocyte aggregates. (R2; CD61+/CD62P+/CD45+), respectively. **B**) The relative number of CD62P+ platelets in the single platelet fraction (R1) was calculated for each sample by setting the M1 marker in the isotype control histogram (turquoise line). Unstimulated platelets from buffy coat platelet concentrates (BC, red line), the PBPC samples (green line), the waste fractions (orange line) and from the target fractions (dark blue line) were analysed (**B1**). Platelets from the waste fraction and from BC were analysed again after stimulation with TRAP (**B2**).

**Figure 5:**
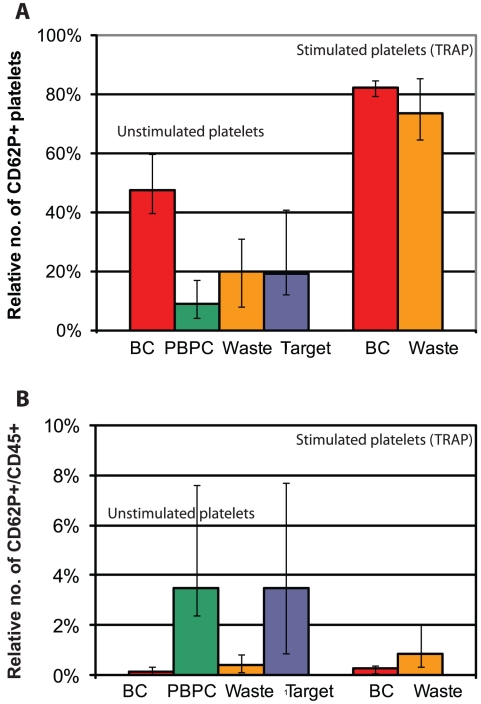
Platelet activation and aggregate formation. Platelet activation based on the surface expression of P-selectin (CD62P). The median relative numbers of single platelets expressing CD62P (**A**) and the median relative numbers of CD61+/CD62P+/CD45+ platelet-leukocyte aggregates (**B**), respectively, are given as the median (range) of 5 individual samples (sample no. 11–15).

Analysis of platelet activation was performed on cells from PBPC samples (n = 5) before and after acoustophoretic separation, using the α-granule membrane protein CD62P (P-selectin) as an activation marker (Figure 4). In flow cytometry analysis, a major population of CD61+ single platelets was observed, with a variable relative number of CD61+/CD62P+ events in each sample. There was a significant increase (p<0.1) in the median relative number of single platelets expressing CD62P in the target (19%, range 12–41%) and waste fractions (20%, range 8–31%), respectively, as compared to the PBPC samples (9.0%, range 4–17%). In control samples (n = 4) obtained from standard buffy coat (BC) platelet concentrates, however, the median relative number of activated platelets (48%, range 40–60%) was significantly higher than in the acoustophoretically separated fractions (p<0.05). Stimulation of platelets with TRAP resulted in significant (p<0.1) increases in the relative numbers of CD62P+ platelets in the waste fractions (median 74%, range 65–86%), which were comparable to activation levels observed in BC platelet concentrates (median 82%, range 79–85%). Activated platelets bound to leukocytes, i.e. CD62P+/CD45+ events that were observed in the PBPC samples, were almost exclusively separated to the target fractions. Acoustophoresis, however, did not increase the formation of platelet-leukocyte aggregates (Figure 5).

## Discussion

The excessive collection of platelets using current centrifugation-based PBPC apheresis presents a problem in PBPC processing, with a negative impact on the performance (yield and purity) of cell product manipulation, such as selection or depletion of specific cell subsets [14], [15], [28], [29]. Furthermore, PBPC apheresis-related co-collection of platelets causes a potentially clinically significant reduction of donor platelet levels, which translates into an increased risk of severe procedure-induced thrombocytopenia. This is especially relevant for patients undergoing PBPC apheresis for autologous transplantation with already reduced pre-apheresis counts due prior cytostatic treatment and mobilizing chemotherapy [10]–[13].

Based on recent developments in the use of acoustic forces in cell separation [20]–[27], we have investigated a micro-chip based acoustophoretic platform for the removal of platelets from PBPC products for retransfusion to the PBPC donor. By applying an acoustic standing wave field on to the continuously flowing PBPC suspension, leukocytes could be almost exclusively separated to the target fraction (98%) whereas platelets were efficiently depleted (89%). The size dependent fractionation of leucocytes versus platelets was accomplished by tuning the acoustic power such that the larger leukocytes (5–20 µm in diameter) [30] were focused into the center of the micro channel while the smaller platelets (2–4 µm in diameter) [31] were left unaffected and remained at the lateral walls (Figure 1B). As could be expected, the few leukocytes that were not focused toward the channel center and thus, lost to the waste fraction, were mainly lymphocytes (5–10 µm in diameter), which are smaller and less acoustically affected than the larger granulocytes/monocytes. However, the total loss of lymphocytes to the waste fraction was minimal and thus would not be of any clinical significance. Furthermore, composition of leukocyte populations was conserved with a comparable median relative number of lymphocytes, CD3+ cells, CD4+ cells, CD8+ cells, CD14+ cells, CD56+ cells, CD19+ cells and CD34+ cells in the PBPC samples and the target fractions.

A possible negative influence of acoustophoresis on cell viability could be excluded as cell viability post sorting was not different from pre-sorting values. Furthermore, colony-forming ability, which is a sensitive assay for functional capacity of early hematopoietic progenitor cells, was not affected by the acoustophoresis procedure.

As previously described, RBC (7 µm in diameter, density 1.100 g/mL) and platelets (2–4 µm in diameter, density 1.058 g/mL) [30], [31] which behave similarly in an acoustic force field can be efficiently separated by adjusting the suspending medium density with cesium chloride [26]. In our study, where the acoustic force was tuned to optimize platelet depletion without loss of target cells, the RBC were distributed to the target fractions, as detected by inspection of cytospin slides (data not shown). No RBC hemolysis was evident by visual inspection of acoustophoresis separated samples. As RBC contamination is generally low in PBPC apheresis products and seldom presents a problem in cell processing or transplantation [10], [11], [13], the issue of RBC was not further addressed in this study.

The alpha-granule membrane protein P-selectin (CD62P) is a widely used marker for detection of platelet activation [32]–[37]. In this study, CD62P was applied for the evaluation of acoustophoresis-induced platelet stress, in a modified flow cytometry assay designed to minimize artefactual activation of the samples [35], [36], [38]. The low level of platelet activation that was seen in collected PBPC apheresis samples (9%) was increased in the acoustophoretically separated target (20%) and waste fractions (19%), respectively, however not to the extent observed in BC platelet concentrates that are routinely used for clinical transfusion. Acoustophoresis separated platelets furthermore showed a preserved activation capacity as indicated by an adequate response to the stimulation with TRAP, which can induce full platelet degranulation without initiating coagulation [32].

Our findings of platelet activation induced by acoustophoretic cell separation were well in agreement with previous reports on platelet activation in standard centrifuge-based cell apheresis technology, where activation levels ranging from 10–30% of the collected platelets are seen with different cell separators. Also in PBPC apheresis, significant platelet activation may be induced as shown by the detection of CD62P positive platelets in the circulation of donors [41]. Considering furthermore the relatively high activation rate of 20–45% in BC platelet preparations as described by others [34], [35], [39], [40] and as confirmed by our data presented herein, it appears unlikely that infusion of acoustophoretically separated platelets would confer a higher risk of thrombotic complications compared to current standard platelet products, which are routinely and safely used for clinical transfusion.

The possible adverse effects, infusing PBPC products containing activated platelets has to be considered. Adhesion of CD62P expressing platelets to circulating leukocytes has been suggested to play an important role in the pathogenesis of inflammatory events [42]. Larger platelet-platelet or platelet-leukocyte aggregates, which are sometimes visible in platelet rich PBPC products, may also present a risk of harmful effects to the recipient. The negative impact of high platelet numbers on target cell yield in immuno-magnetic cell selection has also been attributed to platelet-leukocyte interactions, with coating of target leukocytes by platelets, thus preventing the binding of selection antibody [28]. There is, thus, a clear need for an efficient and gentle method for platelet depletion of PBPC products.

Currently used methods for platelet depletion of PBPC products are based on low speed, successive manual [14], [15] or automated [29] centrifugation which typically result in 80–95% platelet depletion and more than 90% recovery of leukocytes. Similar results on cell separation efficiency were found in this study, using acoustophoresis. However, a direct comparison between the two separation techniques, using clinical scale PBPC samples, would be necessary to draw final conclusions. Current, centrifugation based methods may, despite low g-forces, induce interactions between platelets and adjacent leukocytes. In acoustophoresis, the size dependant fractionation provides an instant physical separation of leukocytes and platelets, thus preventing further aggregate formation, as shown in this study. In a future clinical setting, acoustophoresis has the potential to provide an efficient platelet depletion which, despite an increase in activation level of the few platelets remaining in the target fraction, will result in a markedly reduced total number of activated platelets and also minimize further leukocyte-platelet aggregate formation in PBPC products.

In applications demanding high throughput, several separation channels can be operated in parallel, similar to the approach previously used in microchip-based blood washing where a device comprising eight separation channels in a bifurcation tree was used to increase the flow rate to 0.5 ml/min [43]. Such an approach would open up the possibility for ‘in-line’ platelet depletion of collected PBPC directly following separation in the collection chamber of the apheresis instrument. This would require a flow rate of 1–2 ml/min and is thus technically feasible. The instant physical separation of collected leukocytes and platelets would prevent platelet activation in further handling of the PBPC product and, furthermore, enable to simultaneously re-infuse the acoustophoresis separated platelets to the donor, thus preventing apheresis-induced thrombocytopenia.

In summary, we conclude that the acoustophoresis technique as described herein can be utilized to efficiently deplete PBPC samples of platelets, whilst preserving the target leukocyte fraction, cell viability and progenitor cell colony-forming ability. Acoustophoresis is, thus, an interesting technology to improve current cell processing methods, with the potential to efficiently deplete PBPC products of intact platelets for re-transfusion to the donor, while preserving the number and function of the collected PBPC in a future clinical apheresis setting.

## Materials and Methods

### Ethics Statement

Sampling of patient and donor PBPC products for use in the current study was approved by the Regional Ethical Review Board at Lund University. Written informed consent was obtained from all participants involved in the study.

### Collection of PBPC samples

Samples were obtained from PBPC products collected after standard mobilization treatment of healthy donors (G-CSF, Neupogen; Amgen, Thousand Oaks, CA, USA) and patients (protocol specific chemotherapy + G-CSF). Large volume leukapheresis was performed with a Cobe Spectra (Cobe, Lakewood, CO, USA), using the MNC program, version 7.0. On the day of the leukapheresis, or after a maximum of 24 hours storage, 1 mL of PBPC sample was removed from the collection bag, analysed using a standard automated hematology analyzer (Sysmex KX-21N, Sysmex, Kungsbacka, Sweden), and surplus cells were used for further experimentation.

### Acoustophoresis chip design

The chip was designed to consist of a single acoustophoresis channel dividing at each end of the chip into a trifurcation with one central orifice and the laterally dividing branches joining to form a single side orifice, thus resulting in a chip with two inlets at one end and two outlets at the other (Figure 1). The silicon acoustophoresis channel was made by anisotropic wet etching using Potassium hydroxide (220 g KOH in 550 MilliQ H_2_O) and sealed with a glass lid using anodic bonding. The channel width was set to 380 µm to match half a wave length resonance of 2 MHz. Silicon tubing with an inner diameter of 1/16 inches was glued to the inlets and outlets, to act as docking ports for fluidic tubing.

### Acoustophoresis set up

**Figure 6:**
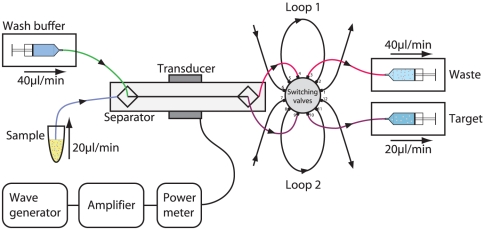
Schematic diagram of the acoustophoresis set up. Two pumps connected to the chip outlets and set in withdrawal mode, and one pump connected to the chip central inlet, infusing wash buffer were responsible for flow control. The PBPC sample was connected to the chip side inlet and entered into the separation channel at a net flow rate set by the three pumps. A switching valve injector was inserted between the chip outlets and the outlet syringe pumps enabling the collection of outlet samples. The ultrasonic standing wave was generated by a piezoelectric transducer operated via an amplifier by a waveform generator setting the resonance frequency to 2 MHz.

The chip was equipped with three syringe pumps (WPI SP210iwz, World Precision Instruments, Sarasota, FL, USA) connected to 5 mL plastic syringes (BD Plastipak Luer-Lok Tip, Franklin Lakes, NJ, USA) to control the flow through the chip (Figure 6). Two pumps were connected via Teflon tubing (VWR Intnl. O.d. 1.54 mm, I.d. 0.05 mm) to the chip outlets and set in withdrawal mode. The third pump was connected to the chip central inlet, infusing wash buffer (Dulbecco's Phosphate Buffered Saline (PBS), pH 7.4; 1% Bovine Serum Albumin (BSA); 2 mM Ethylenediaminetetraacetic acid disodium salt dehydrate (EDTA), all from Sigma-Aldrich). The PBPC sample was connected to the chip side inlet and entered into the separation channel at a net flow rate set by the three pumps. A switching valve injector (25.EPC12W, VICI, Valco Instruments, Houston, TX) was inserted between the chip outlets and the outlet syringe pumps, allowing the intermittent connection of sampling loops (Teflon tubing, 128 µl) to the system and thus, enabling the collection of outlet samples without syringe pump manipulation. The ultrasonic standing wave was generated by a piezoelectric transducer (PZ26, Ferroperm Piezoceramics, Kvistgard, Denmark), which was operated via an amplifier (Amplifier Research 75A250, Southerton, PA, USA) by a waveform generator (Agilent 33220A, Hewlett-Packard Inc., Palo Alto, CA, USA), setting the resonance frequency to 2 MHz. The delivered electric power was monitored by a Bird model 5000-EX digital power meter (Bird Electronic Corporation, Cleveland (Solon), OH, USA) and visual monitoring of the set up was performed using a Nikon SMZ800 microscope (LRI Instrument, Lund, Sweden).

### Fluidics and sampling procedure

The two pumps in withdrawal mode were set to give a flow rate through each of the three exit branches of 20 µl/min. The wash buffer was infused at a flow rate of 40 µl/min, leaving a net flow rate of 20 µl/min (10 µl/min in each side inlet branch) for the PBPC sample. The sampling loops were pre-filled with PBS in order to eliminate air flowing into the system when the loops were set to collection mode. The PBPC samples were diluted 1∶5 in PBS and connected to the side inlet of the chip. The operational parameters were tuned by visual inspection of the outlet trifurcation and set when leukocytes were optimally focused into the central outlet and platelets were distributed to the lateral branches (Figure 1B and Movie S1).The sampling loops were activated and allowed to fill up with separated fluid for 5 minutes. The loops were disconnected and flushed with air to evacuate the central outlet loop (target fraction) and the side outlet loop (waste fraction), respectively. The diluted PBPC samples, the target and the waste fractions were analyzed for blood cell counts (Sysmex KX-21N, Sysmex, Kungsbacka, Sweden).

### Cell morphology

From each PBPC sample, cytospin slides were prepared from target and waste fraction by centrifugation of 5,000–10,000 cells onto glass slides (5 minutes, 750 rpm. Labex Instruments AB, Göteborg, Sweden) onto glass slides. The cells were stained for 5 minutes with May-Grünwald solution (May-Grünwald, Eosin; Histolab Products, Västra Frölunda, Sweden), washed, fixed for 20 minutes in Giemsa (Histolab Products), washed and blotted dry. The cells were evaluated using a Nikon H 55OL microscope.

### Flow cytometry leukocyte analysis

PBPC samples, target and the waste fractions were analysed for CD34−, CD3−, CD3/4−, CD3/8−, CD14−, CD56− and CD19-positive cells, using a four-colour flow cytometer (FACSCalibur; BD, Becton Dickinson, San Jose, CA, USA). Flurochrome-labelled monoclonal antibodies were used in different combinations as follows: anti-CD34 phycoerythrin (PE), anti-CD45 fluorescein isothiocyanate (FITC), peridinin chlorophyll protein complex (PerCP) or allophycocyanin (APC), anti-CD3 FITC, anti-CD4 APC, anti-CD8 PE, anti-CD56 PE, anti-CD19 PE or PerCPCy5, anti-CD14 PE or PerCP (all BD) and anti-CD15 PE (Becton Dickinson, Pharmingen, San Diego, CA, USA). For isotype controls, IgG1 (FITC) and IgG1 (PE) were used (BD). Propidium Iodine (PI, 100 ug/ml, Sigma-Aldrich) was used for dead cell exclusion. 50,000 events were acquired and data were analyzed with CellQuest software (BD).

### Hematopoietic progenitor cell assays

PBPC samples, target and waste fractions were evaluated for their colony-forming ability in hematopoietic progenitor cell assays using standard methylcellulose culture (MACS HSC-CFU media complete with EPO, Miltenyi Biotec GmbH, Bergisch Gladbach, Germany). Cells were plated at a concentration of 1,000 cells/mL and incubated for 14 days (Thermo Forma Steri incubator, 37°C, 5% CO2). Hematopoietic progenitor cell colony forming units (CFU) were enumerated based on standard criteria using an Olympus IX70 microscope.

### Platelet activation

Control samples were obtained from stored (7 days), blood bank platelet concentrates, which were prepared from pooled buffy-coats, suspended in platelet additive solution T-SOL™ (Baxter Healthcare corp., Deerfield, IL, USA) and depleted of leukocytes by filtration according to standard blood bank procedures. Samples were analyzed for platelet cell count (Sysmex KX-21N, Sysmex, Kungsbacka, Sweden). A number of 20×10^6^ platelets from the control samples, the PBPC input samples, the target and the waste fractions were directly suspended in cold Cellfix (0.5%, BD biosciences). A number of 20×10^6^ platelets from the control samples and the waste fractions were incubated with 20 µM TRAP (Thrombine Receptor Activator Peptide, stock solution 200 µM, Sigma-Aldrich), for 20 minutes in room temperature, after which they were suspended in Cellfix as described above. Control and acoustophoresis samples were washed twice (centrifuged at 1200×g, for 5 minutes) in PBS and re-suspended in 1 ml of DBA (PBS+0.2% BSA+0.1% Sodium Azid, Mallinckrodt Baker, Phillipsburg, NJ, USA). Flowcytometric analysis of CD61 (fibrinogen receptor, platelet specific), CD62P (P-selectin, α-granule membrane protein expressed on the surface of activated platelets) and CD45 (leukocytes) was performed. Fluorochrome-labelled monoclonal antibodies (all BD, Biosciences) were used as follows: anti-CD61 FITC, anti-CD62P PE and anti CD45 PerCP. Samples were analysed using a three-color flow cytometer (FACS-Scan, BD Biosciences). 10 000 events were acquired from each sample and data were analyzed with CellQuest software (BD) (Figure 4).

### Presentation of results and statistical analysis

Data are presented as median (range) or as mean ± standard deviation (SD). Statistical analysis was performed using the Wilcoxon signed rank test for paired observations and the Wilcoxon log rank sum test for comparison of two sample groups. For the latter, a two-sided p-value of <0.05 was considered significant.

## Supporting Information

Movie S1 **Movie clip of the separation process taken at the trifurcation.** The larger leukocytes (WBC) have been centered in the channel by the ultrasonic standing wave and are collected in the central outlet, while the smaller platelets (PLT) are less acoustically affected and exit through the side branches.
(AVI)
